# Distribution of enteric glia and GDNF during gut inflammation

**DOI:** 10.1186/1471-230X-11-3

**Published:** 2011-01-14

**Authors:** Georg BT von Boyen, Nadine Schulte, Carolin Pflüger, Ulrike Spaniol, Christoph Hartmann, Martin Steinkamp

**Affiliations:** 1 Department of Medicine I (Gastroenterology), University of Ulm, Ulm, Germany; 2 Department of Gastroenterology, Endocrinology and Metabolism, University of Marburg, Marburg, Germany

## Abstract

**Background:**

The enteric glia network may be involved in the pathogenesis of inflammatory bowel disease (IBD). Enteric glia cells (EGCs) are the major source of glial-derived neurotrophic factor (GDNF), which regulates apoptosis of enterocytes. The aim of the study was to determine the distribution of EGCs and GDNF during gut inflammation and to elucidate a possible diminished enteric glia network in IBD.

**Methods:**

The expression of glial fibrillary acidic protein (GFAP) in colonic biopsies of patients with IBD, controls and patients with infectious colitis was detected by immunohistochemistry and Western blot. Tissue GDNF levels were measured by ELISA.

**Results:**

The expression of GFAP and GDNF in the mucosal plexus is highly increased in the inflamed colon of patients with ulcerative colitis (UC) and infectious colitis. Although the GDNF and GFAP content are increased in Crohn's disease (CD), it is significantly less. Additionally the non-inflamed colon of CD patients showed a reduced GFAP and no GDNF expression compared to controls and the non-inflamed colon of UC patients.

**Conclusions:**

GFAP and GDNF as signs of activated EGCs are increased in the inflamed mucosa of patients with UC and infectious colitis, which underline an unspecific role of EGC in the regulation of intestinal inflammation. The reduced GFAP and GDNF content in the colon of CD patients suggest a diminished EGC network in this disease. This might be a part of the pathophysiological puzzle of CD.

## Background

Chronic inflammatory bowel disease (IBD) is most commonly categorized into Crohn's disease (CD) and ulcerative colitis (UC) and afflicts primarily the distal small and large intestine [1]. At present, there is no definite cure for these diseases and although the etiology of IBD remains unknown there are some new insights into its pathophysiology. Both CD and UC are characterized by a significant increase of proinflammatory cytokines in the gut, which trigger and support the inflammatory processes [2]. Several lines of evidence implicate glial fibrillary acidic protein (GFAP)-positive enteric glia cells (EGC) in regulating the inflammatory response in the gut as well as the integrity of the gut epithelium [3-7]. Genetic ablation of EGCs in mice induced fatal hemorrhagic jejuno-ileitis with histopathological changes closely resembling changes described in human CD [7,8]. Destruction of EGC by auto-immune mechanisms was shown to induce gut inflammation [9]. EGC processes are in close proximity to gut epithelial cells and these cells secrete several mediators implicated in mucosa barrier function. EGCs are producers of transforming growth factor β (TGF- β) that promote intestinal barrier function [10]. Recently it was shown that nitric oxide metabolite S-nitrosogluthatione (GSNO), a novel potent inducer of intestinal barrier function in human colon [11] is secreted by EGCs. Furthermore we could show that EGCs secrete glial-derived neurotrophic factor (GDNF) whose production is increased during intestinal inflammation and could act to protect intestinal epithelial cells from cytokine-induced apoptosis [12-14].

It is postulated that EGC populations and its mediators are perturbed prior to the clinical onset of intestinal inflammation [15]. It remains elusive, if CD [9] and/or UC are indeed characterized by a decreased EGC network and if these changes mark the onset of intestinal inflammation in IBD.

Therefore we investigated the GFAP content as marker for EGCs in the inflamed and non-inflamed intestines of patients with CD and UC in comparison to controls and patients with infectious colitis and postulate a diminished EGC network with reduced GDNF content in the intestines of patients with CD.

## Methods

### Human tissue

The 101 patients enrolled in the study gave their informed consent and the study was approved by the ethical committee of the University of Ulm, which is leaded by Prof. Dr. U. Brückner. The diagnosis of CD and UC was established by using usual criteria [16]. Inflamed and non-inflamed colonic biopsies were taken from 35 patients with CD (15 female/20 male; mean age 31 years; range 21 to 48 years) and from 30 patients with UC (12 female/18 male; mean age 34 years; range 24 to 52 years). 15 patients with CD were in clinical remission (Crohn's Disease Activity Index CDAI < 150) and 20 patients showed clinically a mild flare (CDAI > 150). 10 patients with UC were in remission defined by a Colitis Activity Index (CAI) of 0, whereas the other 20 patients suffered from a flare, CAI > 4. Biopsies were taken during routine ileocolonoscopy only of the left-sided colonic parts, descending colon and sigmoid. The definition of non-inflamed or inflamed tissue was depending on Endoscopic activity using the Simple Endoscopic Score. (SES-CD) [17]. A SES-CD of 0, meaning no signs of active inflammation in a colonic segment defined "non-inflamed". Five biopsies of a defined inflamed or non-inflamed colonic segment (descending colon or sigmoid) were taken for analysis.

The mean duration of CD was 6.5 years and the mean duration of UC was 4.5 years. No patients with CD or UC received biologics. 10 CD patients were treated with azathioprine, one patient with 6-MP and 3 patients with budesonide. 8 patients with UC were treated with azathioprine and 14 patients with mesalamine. The other patients were not treated at the time of study and had only less clinical signs of activity.

As controls healthy colonic biopsies were taken from 26 patients, which underwent a routine screening colonoscopy (11 female/15 male; mean age 53 years; range 48 to 57 years). Colonic biopsies of 10 patients with infectious colitis (Clostridium difficile) served as controls for the inflamed intestines (7 female/3 male; mean age 68 years; range 59-74 years). Tissue GFAP levels were measured in a blinded fashion in patients by Western blot analysis and enzyme-linked immunoabsorbent assay (ELISA).

### Indirect immunofluorescence

Tissue-biopsies were deparaffinized and permeabilized with PBS/0.3% Triton X100. Slides were blocked with 1% bovine serum (Sigma) in PBS. Antibodies against GFAP (Pharmingen, San Diego, USA, mouse) and GDNF (R&D Systems, Wiesbaden, Germany, goat) were used at dilutions of (1:100) and incubated overnight at 4°C. After washing in PBS/0,1% Tween 20, the slides were incubated with the appropriate secondary antibodies: cy3 coupled goat anti-mouse IgG (DPC Biermann, Bad Nauheim, Germany) and Alexa 488 coupled rabbit anti-goat IgG (Sigma). Dilutions of the secondary antibodies were 1:800. After washing in PBS, slides were embedded in glycerol gelatine. Control labelling was performed with omission of the first antibodies to ensure that there was no unspecific labelling of cells.

Tissue-biopsies were analyzed using a Leica confocal laser-scanning microscope. Single optical sections were recorded under conditions, which exclude cross-activation of the individual signal channels.

### Histological analysis

Every Immunolabelled biopsies of 35 CD, 30 UC, 10 infectious colitis patients or 26 control persons were used for evaluating the expression of GFAP and GDNF in EGCs.

### Western blot analysis

Proteins were extracted from three pooled biopsies in 100 μl of lysis buffer (1% Triton X-100, 150 mM NaCl, 20 mM, Tris-HCL pH 7.5, 2 mM EDTA, 1 × protease inhibitor cocktail) for 30 min at 4°C, followed by centrifugation for 10 min at 12,000 r.p.m. at 4°C.

All protein samples were normalized to the total protein content (Bradford reagent; Bio-Rad Laboratories, Hercules, CA, USA), and comparable loading, as well as protein transfer after blotting, was confirmed through detection of Actin as internal control. Protein was loaded on a 10% SDS gel, separated and blotted. After blocking with 5% skim milk, primary antibody (glial fibrillary acidic protein (GFAP) Pharmingen, San Diego, USA, mouse) was incubated overnight at 4°C. Membranes were washed twice with TBS-T, incubated with secondary antibody (anti-mouse HRP (1:5000; Dako)), followed by subsequent washing steps and visualized with ECL (Amersham Biosciences, Freiburg, Germany).

Western blot bands were analysed by scanning densitometry. Multiple exposures of each blot were used to obtain grey scale images and the GFAP (Pharmingen) immunoreactive bands were quantified with the Quantity One Analysis program from Bio-Rad. The data presented in the figures are expressed as x-fold above controls.

### Enzyme-linked immunoabsorbent assay (ELISA) of colonic tissue

GDNF levels in colonic tissue of patients and controls were assayed using an ELISA of Promega as described before (14). The detection limit of this assay was about 30 pg/ml for GDNF.

### Data analysis

All data given in the text and figures are expressed as mean values ±SEM. The data were analyzed using non-parametric two-tailed Mann-Whitney U test with p≤0.05 considered as an indicator of significance.

## Results

### GFAP and GDNF is increased in gut inflammation

**Figure 1:**
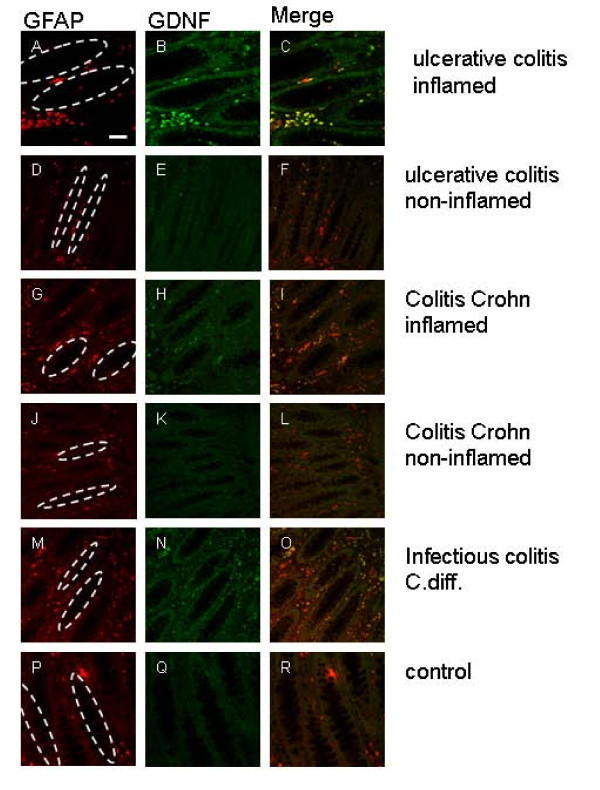
**Biopsies of the inflamed and non-inflamed colon of patients suffering from CD, UC, infectious colitis and controls were double immunolabelled with anti GFAP (A,D,G,J,M,P red) and anti-GDNF (B,E,H,K,N,Q green) antibodies and were analyzed by optical sectioning using a confocal microscope**. The dotted ovals indicate the position of epithelial crypts. Both antigens, GFAP (A) and GDNF (B) can be detected highly in the intestinal wall of the inflamed colon of UC (A,B)-, infectious colitis (M,N)- and to a smaller extent in CD patients (G,H). The merged images (C,I,O) reveal an almost complete overlap of both immunoreactivities (yellow). Only few GFAP-positive cells (G) display no GDNF immunoreactivity (H, I) in the non-inflamed colon of CD patients and the control section (P-R), whereas in non-inflamed tissue of UC patients GFAP-positive EGCs (D) showing slight GDNF expression (E). GFAP-positive EGCs (A,D,G,J,M,P) are positioned in the mucosal plexus in close vicinity to the epithelium of the colon. Scale bars, 50 μm.

EGCs in gut biopsies were identified by immunocytochemical labelling of GFAP and GDNF, which reveals the enteric glia structures of the mucosal plexus. In the inflamed intestines of patients with UC a high increase of both GFAP-positive EGCs and GDNF was seen (Figure 1A-C) in comparison to control (Figure 1P-R). The overlay demonstrates the GDNF expression of GFAP-positive EGCs.

The non-inflamed gut biopsies of UC patients seemed to be characterized by a more dense enteric glia network (Figure 1D-F) than controls (Figure 1P-R), but significantly less than the inflamed sections (Figure 1A-C).

Patients with colitis Crohn also had an increase in GFAP and GDNF expression in inflamed colonic biopsies (Figure 1G-H), but less impressive than patients with UC (Figure 1A-C). Colonic parts without inflammation in patients with colitis Crohn (Figure 1J-L) showed a reduced labelling of GFAP and no expression of GDNF in comparison to non-inflamed gut structures of patients with UC (Figure 1D-F).

The biopsies of patients with infectious colitis (Clostridium difficile) was characterized by a impressive increase of GFAP- and GDNF-positive structures (Figure 1M-Q) similar to the inflamed gut biopsies of patients with UC (Figure 1A-C).

The biopsies (Figure 1A-R) revealed, that the GFAP-positive EGCs, which highly express GDNF, are positioned in the mucosal plexus in close vicinity to the mucosal epithelium.

### (Semi-)Quantification of GFAP expression in the inflamed intestines

**Figure 2:**
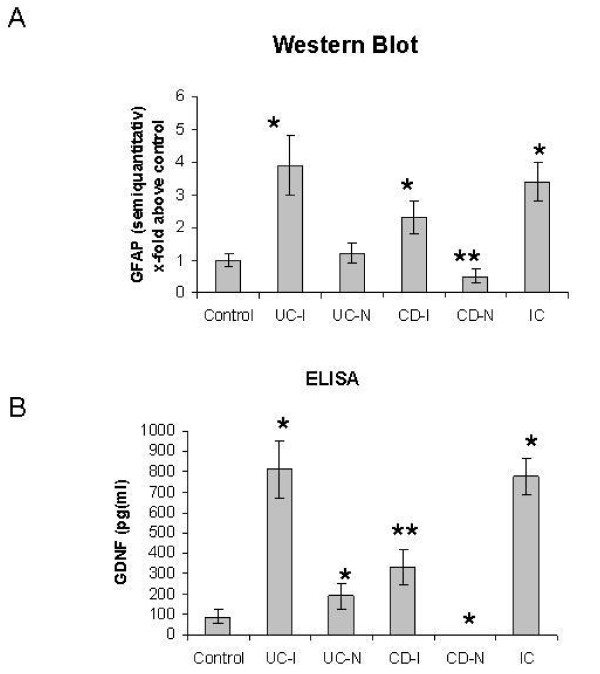
**Tissue GFAP levels in clinical biopsies**. (A). Western blot analysis of tissue extracts from controls, inflamed UC (UC-I), non-inflamed UC (UC-N), inflamed CD (CD-I), non-inflamed CD (CD-N) and infectious colitis (IC) using anti-GFAP antibodies. (A) shows the densitometric analysis of GFAP expression in the colon tissues of the different clinical groups; the data are expressed as x-fold above control. * indicates a significant increase of GFAP in tissue samples of inflamed intestines compared with control (p < 0.05). ** indicates a significant reduction of GFAP in CD-N compared to control and UC-N (P < 0.05). (B) GDNF concentrations (pg/ml) in the colon biopsies of the different clinical groups. *Significantly different from control (P < 0.05). **Significantly different from UC-I and IC (P < 0.05)

Western blot analysis of extracts from colonic biopsies of controls revealed a weak basal expression of GFAP (Figure 2A). However, in the inflamed colon of patients with UC GFAP expression was upregulated 4-fold above control levels (Figure 2A). Low basal expression levels of GFAP were detected in non-inflamed colonic tissue of UC patients. Although an increased GFAP content was observed in the inflamed gut tissue of CD patients, it was less than in biopsies of UC patients. The non-inflamed intestines of CD patients were characterized by a low GFAP expression, which was less than controls or in the non-inflamed gut tissue of patients with UC.

**Figure 3:**
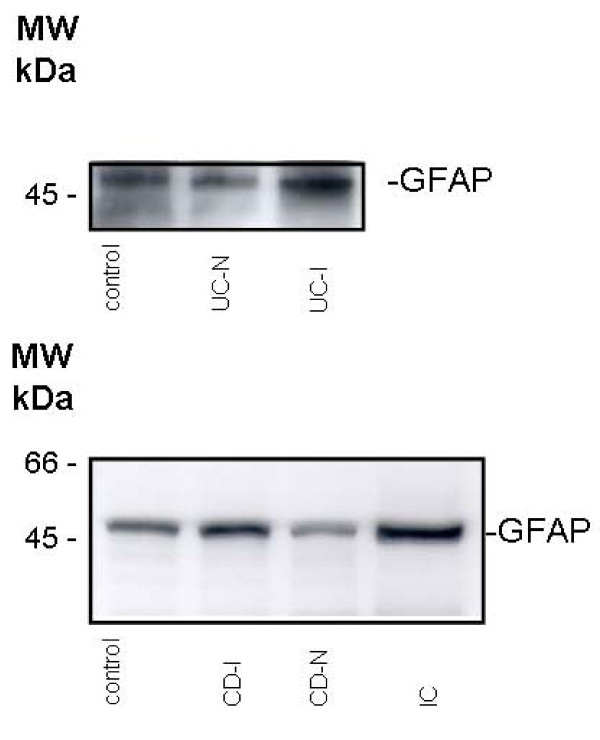
**An exemplarily Western blot analysis of colonic biopsies**. One lane represent four biopsies from control, inflamed UC (UC-I), non-inflamed UC (UC-N), inflamed CD (CD-I), non-inflamed CD (CD-N) and infectious colitis (IC).

The GFAP expression of the inflamed intestines in patients with infectious colitis showed nearly the same impressive GFAP content, about 4-fold in comparison to control, like gut inflammation in patients with UC (Figure 2A). An exemplarily Western-blot of colonic biopsies is shown in Figure. 3.

### Increased levels of GDNF in the inflamed gut

In the gut of control persons we found a basal expression of GDNF (89 ± 32 pg/ml). In the inflamed colon of IBD patients a significant increase of GDNF content was observed as illustrated in Figure 2B. A 2-fold upregulation of GDNF protein was detected in non-inflamed colonic tissue of UC patients (190 ± 66 pg/ml). The highest increase of GDNF content could be observed in the inflamed colon of patients suffering from UC (812 ± 138 pg/ml, p < 0.05), and in patients suffering from infectious colitis (775 ± 93 pg/ml, p > 0.05).

Although the GDNF was upregulated in the inflamed gut tissues of CD (335 ± 86 pg/ml), it was statistically less than in UC patients or in infectious disease (p < 0.05). The non-inflamed tissue of CD patients demonstrate no GDNF expression by ELISA (<30 pg/ml), which is significantly less than control (p < 0.05) (Figure 2B).

## Discussion

CD and UC are characterized by an inflammation of the intestines, which is accompanied with an extensive release of proinflammatory cytokines [2]. Recent studies with cultured S100-positive EGCs revealed an induction of GFAP expression upon cytokines stimulation [13]. However, the number and distribution of GFAP-positive EGCs in the inflamed and non-inflamed gut of IBD patients is controversially discussed and only few data exist on EGCs in the intestines of IBD patients and during gut inflammation [9,14]. Here we determined the distribution of GFAP-positive EGCs and GDNF in the inflamed and non-inflamed colon of patients with CD, UC and infectious colitis.

It is speculated that the enteric nervous system (ENS) is disturbed in chronic inflammation of the gut [3-9]. Previous data showed an increase of GDNF and GFAP in sections of patients with CD [14]. Our data demonstrate, that in the inflamed tissue of patients with UC, both, GFAP-positive EGCs and GDNF is also highly increased. As the same is observed in the colon biopsies of patients suffering from infectious colitis, increased EGCs secreting GDNF might be not a specific phenomenon in IBD, but a general reaction of the mucosal ENS during gut inflammation.

Furthermore, we had demonstrated that GDNF secretion is increased in the inflamed mucosa of CD and that GDNF acts anti-apoptotic on intestinal epithelial cells [12,14]. EGCs serve as the main source of mucosal GDNF and a loss of EGCs [14], lead to a severe inflammation of the gut by a disruption of the mucosal barrier [18]. A defect EGC network is postulated for CD. This is underlined by our findings. Although GDNF - anti-apoptic agent for epithelial cells- and GFAP is increased in the inflamed gut of CD, it is significantly less than in the inflamed intestines of UC patients or patients with infectious colitis. Interestingly, in the non-inflamed tissue of CD patients, GDNF could be not detected in the range of the used ELISA (>30 pg/ml), as sign of a reduced GDNF secretion, maybe caused by a disturbed EGC network. The diminished GFAP expression in the non-inflamed and in the inflamed tissue of CD patients might be an indicator for a chronic dysfunction of EGCs.

As loss of EGCs lead to gut inflammation [7,8] and several animal models demonstrate EGCs and their secreting factors (GDNF, β TGF- β, GSNO) as essential tools for maintaining the integrity of the gut [9-11,14], it might be speculated that the reduced EGC network of CD patients is part of the pathophysiological puzzle in this disease.

It is imaginable that innate defects of the enteric glia network lead to a leaky mucosal barrier, which influences the adaptive immune system [6,19]. This might be a speculative process in developing CD. Otherwise a chronic transmural inflammation like in CD might lead to a diminished enteric glia network, which reacts insufficient during mucosal inflammation. The loss of EGCs and their protecting substances like GDNF, TGF- β and GSNO lead to the failure of the mucosal barrier and exaggerate intestinal inflammation. The inflammation in UC is localized in the luminal parts of the intestines, e.g. in the mucosa. Thus, there might be no relevant defects in the enteric glia network of UC patients, which is underlined by our results with a high secretion of GDNF during gut inflammation.

## Conclusions

In conclusion our data show that the protective feed back loop of GFAP-positive EGCs, secreting GDNF to regenerate an intact barrier function is not specific for IBD but an unspecific reaction of the enteric glia network during gut inflammation. Furthermore the intestines of CD patients are characterized by a reduced number of EGCs with a diminished secretion of GDNF during inflammation and might be a part of the pathophysiological processes in CD. This underlines that CD and UC are different diseases.

## Abbreviations

CD: Crohn's disease; EGC: enteric glia cell; ELISA: enzyme-linked immunoabsorbent assay; ENS: enteric nervous system; GDNF: glial-derived neurotrophic factor; GFAP: glial fibrillary acidic protein; GSNO: S-nitrosogluthatione; IBD: inflammatory bowel disease; TGF- β: transforming growth factor β; UC: ulcerative colitis.

## Competing interests

The authors declare that they have no competing interests.

## Authors' contributions

GvB and MS treated the patients in the IBD outpatient clinic and performed the colonoscopies. GvB drafted the manuscript. NS and CP collected the data of the patients and performed the microscopically analysis. US and CH carried out the western blot analysis, indirect immunofluorescence and ELISA. All authors participated in the design of the study and GvB performed the statistical analysis. All authors read and approved the final manuscript.

## Pre-publication history

The pre-publication history for this paper can be accessed here:

http://www.biomedcentral.com/1471-230X/11/3/prepub
